# Immunological and Virological Benefits Resulted from Short-Course Treatment during Primary HIV Infection: A Meta-Analysis

**DOI:** 10.1371/journal.pone.0082461

**Published:** 2013-12-06

**Authors:** Jingjing Chen, Xiaoxu Han, Minghui An, Jing Liu, Junjie Xu, Wenqing Geng, Yangtao Ji, Hong Shang

**Affiliations:** Key Laboratory of AIDS Immunology of Ministry of Health, Department of Laboratory Medicine, The First Hospital of China Medical University, Shenyang, Liaoning, China; Istituto Superiore di Sanità, Italy

## Abstract

**Objectives:**

To assess the potential immunological and virological effects that result from short-course antiretroviral treatment during primary HIV infection (PHI). And to investigate whether treatment initiation time, treatment duration and follow-up time after treatment interruption would affect these post-treatment immunovirological outcomes.

**Methods:**

We systematically searched PubMed, Cochrane Library (to September 2013) and retrieved conference abstracts for studies regarding effects of early treatment during PHI on CD4 count and viral load (VL). Using the method of calculating weighted mean differences with Stata11.0, we conducted meta-analyses on the effect of early treatment on CD4 count and VL. Then we performed subgroup analyses by follow-up time after treatment interruption, treatment initiation time and treatment duration. Baseline immunovirological characteristics were also analyzed to account for potential bias.

**Results:**

Compared to the untreated arm, treatment during PHI not only increased CD4 count by 85.92 cells/μl but also lowered viral load by 0.30 log copies/ml within one year after treatment interruption. However, the benefits declined gradually, reaching no significance 12-24 months after treatment interruption. Baseline immunovirological characteristics and sensitivity analyses of randomized controlled trials indicated that the benefits mentioned above were underestimated. Extending treatment duration beyond 12 months did not increase efficacy.

**Conclusions:**

Short-course treatment during PHI was associated with immunological and virological benefits which last for at least one year after treatment interruption. The conclusions from our study would help the decision-making in the clinical management of PHI.

## Introduction

Early responses to HIV-1 infection are important factors determining disease progression [1]. Primary HIV infection (PHI) may provide the optimal time for intervention that could last long. However, several critical questions remain unanswered: should the HIV-infected patients be treated during PHI? How early should the treatment be initiated? How long should the treatment last? Current guidelines remain vague on the management of this unique stage of infection. For example, treatment recommendations from the United States Department of Health and Human Services (DHHS) consider treatment should be offered to acute or recent HIV infection, although definitive data are lacking [2]. Treatment recommendations from European AIDS Clinical Society (EACS) hold opinions that treatment should be considered if there is severe illness or prolonged symptoms (especially CNS symptoms) [3], while treatment recommendations from World Health Organization (WHO) don’t contain information on what to do with primary HIV infection [4]. 

Treatment guidelines remain vague mainly due to the lack of definitive conclusions on post-treatment immunological and virological benefits of short-course treatment during PHI. As CD4 count and viral load are two main indicators of HIV disease progression, most studies evaluated the effect of treatment during PHI with these two markers. However, it is still controversial whether short-course highly active antiretroviral treatment (HAART) during PHI affects subsequent CD4 count and viral load. Some studies reported that CD4 cell counts did not differ between the early treated group and the untreated group when patients were followed for 33 weeks [5] or three years [6] after treatment interruption, but these findings could not be confirmed by others [7]. As for HIV viral load, some studies found associations between early treatment and lower viral set point after treatment [5,8], which were not supported by others either [9,10,11]. The conflicts between studies may be due to variations in the follow-up length after treatment, the treatment initiation time, the treatment duration, or the lack of statistical power because of small sample sizes. 

Meta-analysis is capable of solving problems with inconsistent results, both by increasing sample size to increase statistical power, and by reducing bias caused by specific population in individual studies. The present study aimed to assess the potential immunological and virological effects that result from short-course HAART during PHI. We also explored whether treatment initiation time, treatment duration and follow-up time after treatment interruption would affect these post-treatment immunovirological outcomes. 

## Methods

### Search strategy

We systematically searched Pubmed, Cochrane Library to September 2013, with no language restriction, for studies regarding effects of treatment during PHI on CD4 count and viral load. We used a search method of combining the MeSH terms and relevant key words to avoid any missing. We used term HIV for human immunodeficiency virus. MeSH terms and key words pertaining to antiretroviral treatment included drug therapy, ART, antiretroviral therapy, and antiretroviral treatment. Terms pertaining to viral load were viral load, viremia, RNA viral, set point. CD4 represented CD4 cell count. Terms related to primary infection included acute infection, recent infection and primary infection. We also searched abstracts of Conferences on Retroviruses and Opportunistic Infections (CROI, 1997-2013) and International AIDS Society Conferences (2001-2012). 

### Study selection

Title and abstract review of all searched articles was completed by 2 of the authors (Jingjing Chen and Minghui An) to identify relevant studies on the topic of treatment during PHI. Then full texts of relevant articles were independently reviewed by 2 of the authors (Jingjing Chen and Xiaoxu Han) to determine eligible studies. 

Studies met the following criteria were included in the meta-analysis: (1) Studies contained patients treated and untreated during PHI (either acute infection, recent infection, or both), no matter randomized controlled trials (RCT) or not. (2) The treatment regimen should be HAART rather than monotherapy which is not so effective (3). Studies that provided the mean, standard deviation (SD) values of CD4 count, viral load or viral set point both at baseline and after treatment interruption, and the sample sizes of both groups (4). We included the comprehensive articles for further analysis if dataset from the same cohort had been published more than once. And we chose the published studies if conference abstracts coincided with published ones. 

The criteria for exclusion are as follows: (1) Studies with chronic infected patients as controls. (2) One-arm studies containing only treated patients without untreated controls (3). Studies conducting treatment continuously without interruption. 

### Data extraction

A standardized data extraction form was used for recording information from eligible studies. We extracted the mean and SD values of CD4 count and viral load in both groups at baseline and after treatment interruption, and the sample sizes of both groups. When the SD values were not reported in individual studies, they were calculated from stand error, 95% confidence interval (95% CI), or exact p values. Median and interquartile range (IQR) values were also used to acquire most of the information of CD4 count and viral load at baseline. Attempts were made to contact authors to request additional data when there was still no enough information. In addition, we also collected information about first authors, years of publication, study locations, cohorts, study designs, sample sizes of the studies, treatment initiation time, treatment duration, treatment regimens and follow-up time after treatment interruption. Two reviewers (Jingjing Chen and Xiaoxu Han) extracted the data independently, and disagreements were reconciled by discussions. 

### Statistical analysis

STATA11.0 software was used to perform the following analyses. As outcome variables were continuous, analyses were therefore performed by the method of weighed mean differences (WMD). Statistical heterogeneity was measured using the Q statistic (p<0.10 indicating a statistically significant heterogeneity) and quantified using the I^2^ index which evaluates the proportion of heterogeneity in total variation. I^2^ values of 25%, 50%, and 75% correspond to cut-off points for low, moderate, and high degrees of heterogeneity. Random effects model analysis was carried out to estimate summary statistics if heterogeneity existed, otherwise fixed effects model was used. Furthermore, we did subgroup analyses stratified by follow-up time after treatment interruption, treatment initiation time and treatment duration. Begg's and Egger's tests were used to assess publication bias (p<0.05 indicating a statistically significant publication bias).

Sensitivity analysis was performed by three methods to examine the impact of methodological quality, statistical models and types of study design on the results: (1) Leave-one-out to repeat analyses to test the influence of single studies on the summary effect; (2) Reanalyze under different effects models; (3) Reanalyze data categorized by study design (whether RCT or not). 

## Results

### Search and identification of eligible studies

**Figure 1:**
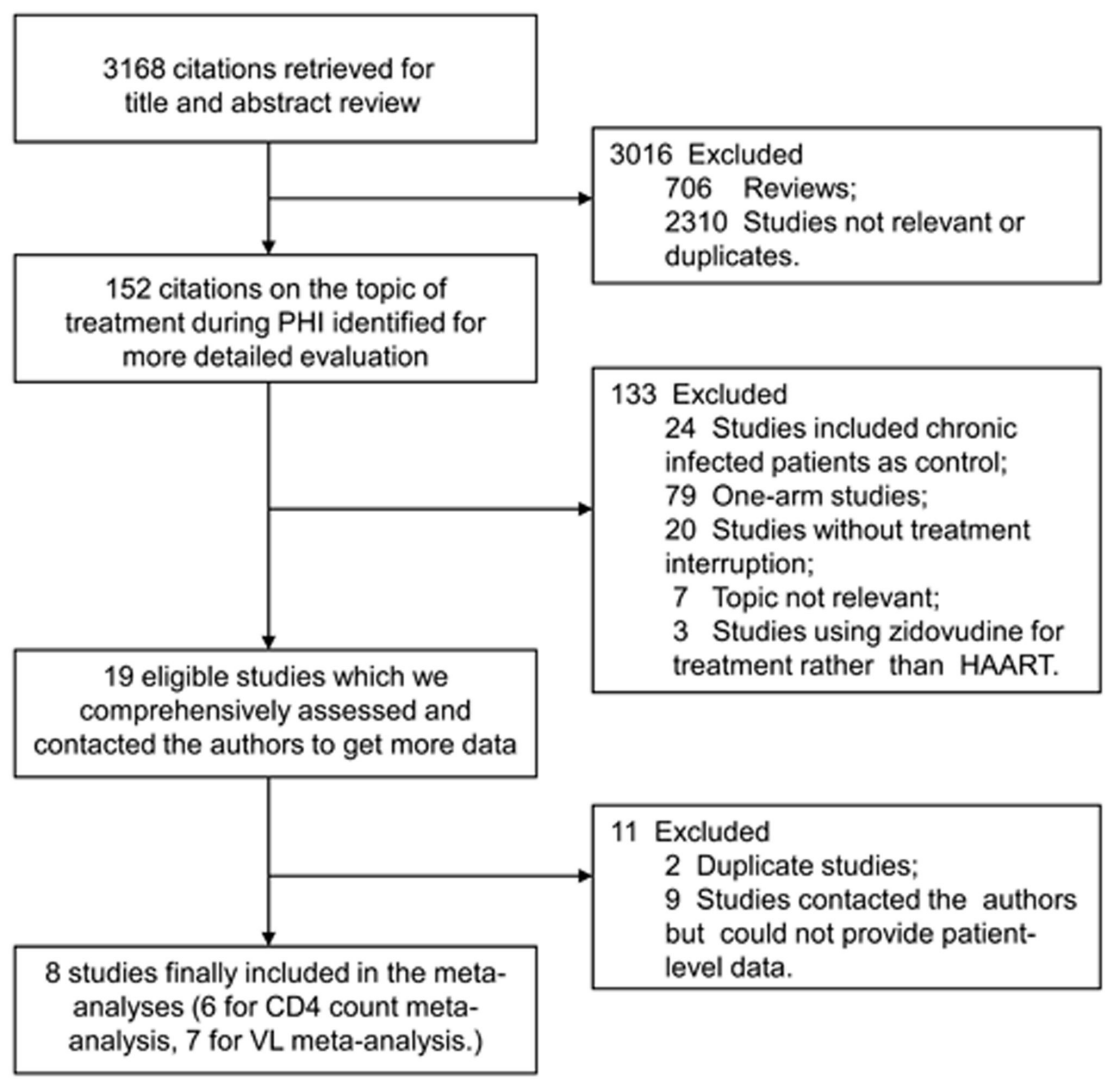
Flow diagram of search, review and data extraction.

As shown in Figure 1, a total of 3168 citations were obtained according to the designed search strategy as described in Methods. Among them, 152 articles on the topic of treatment during PHI were selected for further evaluation, and 19 eligible articles were identified in the second round evaluation. Finally, 8 out of the 19 eligible studies were enrolled in the final analysis after excluding duplicates and those that have no patient-level data. Table 1 summarizes characteristics of eligible studies that met the selection criteria (excluding duplicates).

**Table 1:** Characteristics of eligible studies.

Author, year of publication	Study location (cohorts)	RCT or not	N Treated	N Control	Treatment initiation time	Treatment regimen	Treatment duration	Follow-up time after treatment
Hogan, C. M. 2012 [12]	US, Peru (ACTG A5217)	Yes	39	40	Within 6 months after infection, but beyond acute infection	HAART	36w	36w
Grijsen, M. L. 2012 [13]	Netherlands (Primo-SHM)	Yes	76	36	Acute infection	HAART	24w or 60w	36w
von Wyl, V. 2011 [8]	Switzerland (SHCS)	No	33	32	Acute and recent infection (Time from infection to HAART: median 1.6m, IQR 1.4-1.8m)	HAART	18.2m (15.6-20.5)^a^	Up to 18m
Koegl, C. 2009 [9]	Germany (Prime -DAG, Ac-DAG)	No	100	56	Within 12 weeks after seroconversion	HAART	9.5m (2.1-28.7)^b^	17.3m (1.0-40.4)^b^
Seng, R. 2008 [6]	France (PRIMO and SEROCO)	No	170	123	Within 3 months after PHI diagnosis (Time from infection to HAART: median 38d , IQR 31-52d)	HAART	19m (17-31)^a^	21m (12-36)^a^
Pantazis, N. 2008 [10]	Europe, Austria, Canada (CASCADE)	No	147	675	Within 6 months after seroconversion	HAART	<6m:3m(1.7-4.5)^a^ 6-12m:9.1(7.9-10.9)^a^ >12m:22.4(16.3-38.3) ^a^	Median:15.2m
								Median:9.1m
								Median:11.2m
Fidler, S. 2007 [7]	UK (SMH, CASCADE)	No	81	97	Within 6 months after seroconversion	HAART	3.3m (2.8-4.4) ^a^	2.45y (2.03-3.4)^a^
Desquilbet, L. 2004 [14]	France (PRIMO and SEROCO)	No	58	116	Within 3.5 months after infection (Time from infection to HAART: median 45d , IQR 34-70d)	HAART	Median:17.3m	10.2m (0.9-47.5)^b^
Fidler, S. ^c^ 2013 [15]	UK (SPARTAC)	Yes	243	123	Within 6 months after seroconversion	HAART	12w or 48w	Median:4.2y
Mingrone, H.^c^ unpublished [16]	Argentia	No	12	41	Within 6 months after seroconversion	HAART	72w	72w
Prazuck,T.^c^ unpublished [17]	France	No	6	41	During acute symptomatic primary infection	HAART	NA	NA
Steingrover, R.^c^ 2010 [18]	Netherlands (ACH, HMF)	No	32	250	Within 180 days of seroconversion (Time from seroconversion to HAART: Median 3.9w, IQR 1.8-9.4w,)	HAART	86w (45-139)^a^	3.9y (2.5-5.6)^a^
Lampe, F. C. ^c^ 2007 [19]	Ten countries (Quest, CASCADE)	No	79	385	Within 6 months after start of symptoms (Time from symptoms to HAART: median 18d, range -8-118d)	HAART	2.6y (1.9-5.8)^b^	24w
Hecht, F. M. ^c^ 2006 [20]	US, Canada (AIEDRP)	No	58	337	within 6 months after seroconversion (Time from infection to HAART: median 5w)	HAART	Median:1.5y	Through week 72
Streeck, H. ^c^ 2006 [21]	Germany	No	12	8	Acute infection (Time from infection to HAART: median 25d)	HAART	24w	6m
Jansen, C. A.^c^ 2005 [22]	Netherlands	No	5	6	Within weeks after start of symptoms (Time from seroconversion to HAART: Median 1w)	HAART	53w (22-98) ^b^	NA

Abbreviations : w, weeks; m, months; y, years; NA, not applicable.

^a^ Median and IQR of treatment duration or follow-up time after treatment interruption;

^b^ Median and range of treatment duration or follow-up time after treatment interruption;

^c^ Eligible studies but did not enter the final meta-analyses due to unavailable data.

### Effects of short-course treatment during PHI on post-treatment CD4 count and viral load

**Figure 2:**
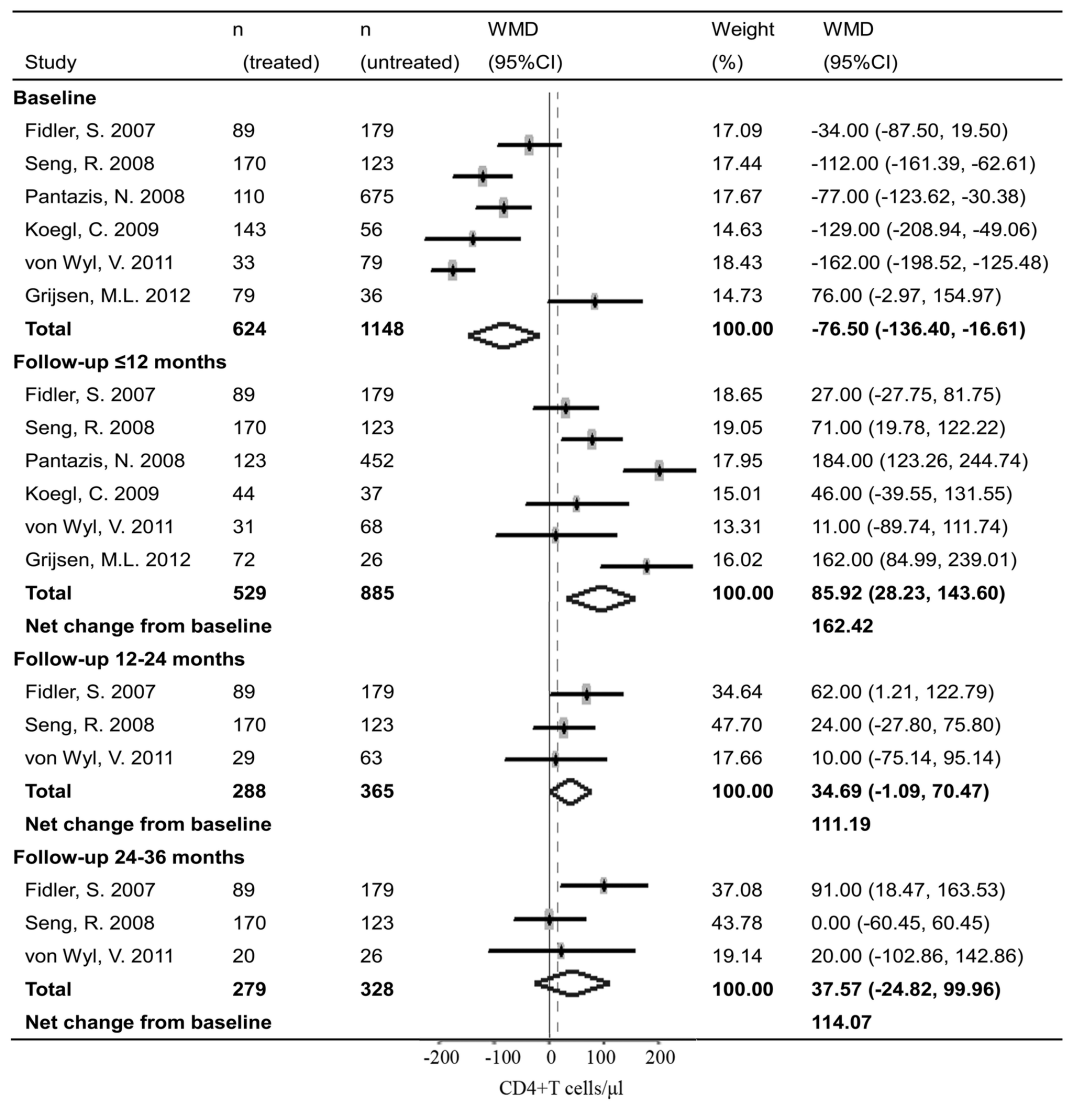
Forest plots of meta-analyses on impact of short-course treatment during PHI on post-treatment CD4 count. Dots represent the individual study estimates; boxes, study weights; and lines, 95% CIs.

**Figure 3:**
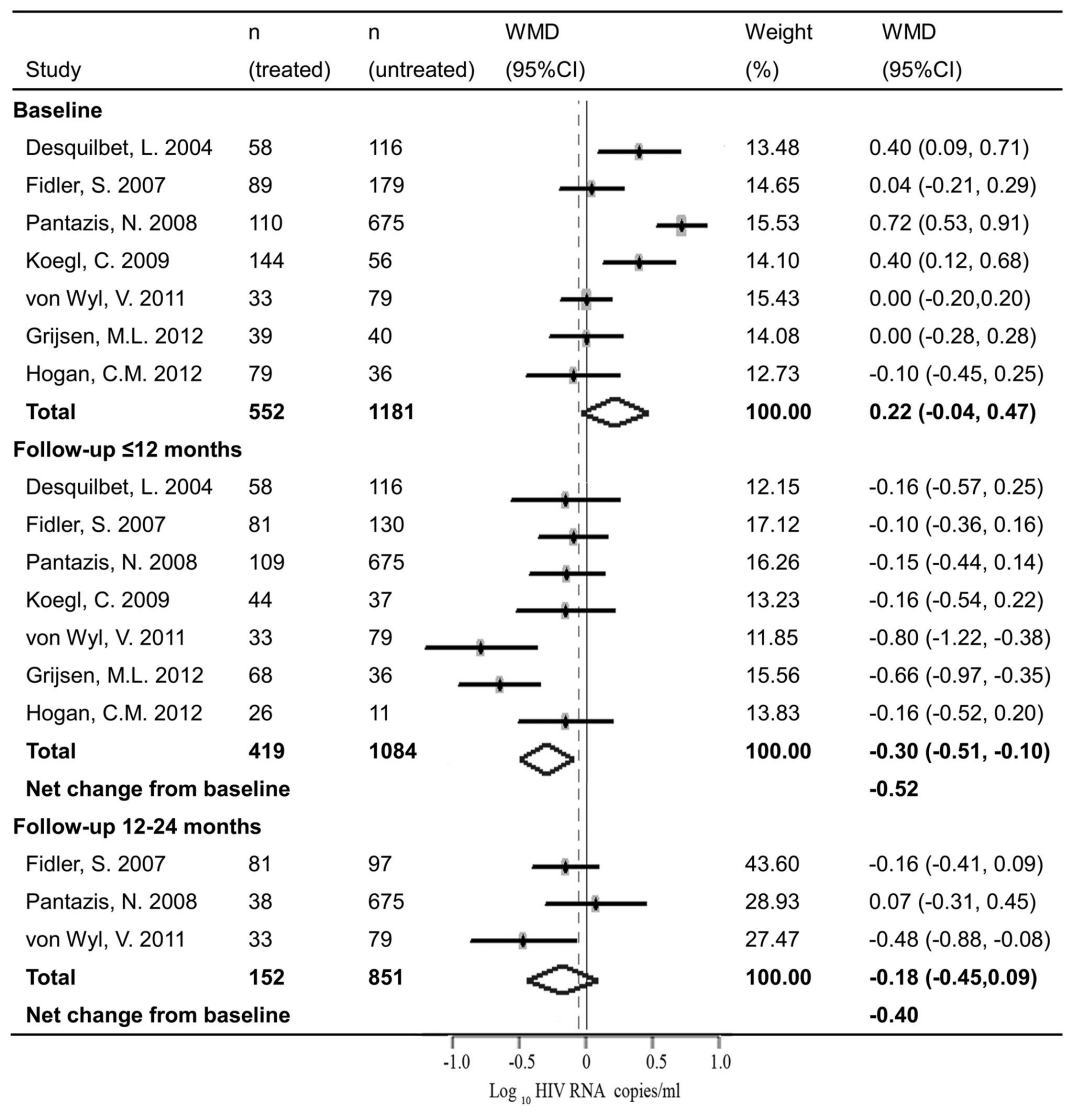
Forest plots of meta-analyses on impact of short-course treatment during PHI on post-treatment viral load. Dots represent the individual study estimates; boxes, study weights; and lines, 95% CIs.

Analyses from pooled studies showed that short-course treatment during PHI had significant immunovirological benefits. Early treatment during PHI increased CD4 count by 85.92 cells/μl (95% CI: 28.23, 143.60, Figure 2) and decreased plasma viral load by 0.30 log copies/ml (95% CI: -0.51, -0.10, Figure 3) compared to the untreated controls within one year after treatment interruption. However, the benefits derived from early treatment could not be sustained and declined over time. The benefit on CD4 count declined from the initial 85.92 cells/μl (in less than 12 months) to 34.69 cells/μl (12-24 months) and 37.57 cells/μl (24-36 months after treatment interruption). And the benefit on viral load dropped from 0.30 log copies/ml (in less than 12 months) to 0.18 log copies/ml (12-24 months after treatment interruption). Of note, statistically, there was no significant difference between groups in CD4 count and viral load after 12-24 months.

Baseline characteristics of CD4 count and viral load between groups were also analyzed as they may influence the immunological and virological benefits. Aggregated findings indicated that there were significant differences between groups in baseline CD4 count and viral load. Compared with the untreated patients, those treated during PHI had lower baseline CD4 count (WMD = -76.50 cells/μl, 95% CI: -136.40, -16.61, Figure 2) and higher baseline viral load (WMD = 0.22 log copies/ml, 95% CI: -0.04, 0.47, Figure 3). After baseline correction, the net changes of CD4 count and viral load derived from early treatment were much greater than those without baseline correction.

Early treatment remained associated with immunological and virological benefits when we used the leave-one-out method and different effects models to repeat the meta-analysis. Results remained similar when studies with monotherapy [11,23] were included. We also performed sensitivity analyses stratified by study design. The differences between groups in CD4 count and viral load from RCT trials were 162.00 cells/μl (95% CI: 84.99, 239.01) and -0.42 log copies/ml (95% CI: -0.91, 0.07) respectively. By contrast, differences in CD4 count and viral load from non-RCT trials were 71.48 cells/μl (95% CI: 9.30, 133.65) and -0.25 log copies/ml (95% CI: -0.47, -0.02). The immunological and virological benefits from RCT trials seemed to exceed those from non-RCT trials. Begg's and Egger's test showed negligible publication bias among studies. 

### Effects of treatment duration on immunovirological outcomes

Based on information of CD4 count and viral load around 12 months after treatment interruption, we showed that increasing treatment duration beyond 12 months was of little help in strengthening the immunological and virological benefits. Patients with treatment duration (or median treatment duration) less than 12 months yielded comparable outcomes to those treated for over 12 months (Table 2). Amongst patients treated for less than 12 months, the CD4 count was 102.46 cells/μl higher, and the viral load was 0.33 log copies/ml lower than those of the untreated patients. While for those treated for over 12 months, the differences between groups in CD4 count and viral load were 107.81 cells/μl and 0.26 log copies/ml respectively. 

**Table 2:** Impact of treatment duration on post-treatment CD4 count and viral load.

	Treat duration	N(treated)	N(untreated)	WMD	95% CI
CD4 count	≤12 months	240	694	102.46	22.63, 182.29
	>12 months	289	669	107.81	22.02, 193.60
Viral load	≤12 months	299	968	-0.33	-0.59, -0.06
	>12 months	158	827	-0.26	-0.50, -0.02

## Discussion

The present study is the first meta-analysis evaluating the immunological and virological impacts of short-course treatment during PHI. We found that short-course treatment during PHI increased CD4 count by 85.92 cells/μl and lowered viral load by 0.30 log copies/ml within one year after treatment interruption. The immunovirological benefits declined gradually after treatment interruption, reaching no statistical significance after 12-24 months. Moreover, extended treatment duration beyond 12 months did not show additional benefits. 

However, the benefits derived from early treatment mentioned above may be underestimated, as most of the enrolled studies are observational ones that are susceptible to recruitment bias and loss of follow-up. Our analysis demonstrated that the early treated patients had higher baseline viral load and lower baseline CD4 cell counts. The disequilibrium in baseline characteristics between groups is one reason that the immunovirological benefits may be underestimated [6,18]. After baseline correction, net benefits derived from early treatment were greater. Sensitivity analyses which indicated that the estimated immunovirological benefits from RCT trials seemed to be larger than those from non-RCT trials also supported this underestimation. Moreover, the dropout rate as disease progressed to treatment (re)initiation was higher in the untreated group compared to the early treated group [12,13]. This may lead to an overestimation of CD4 count and an underestimation of viral load in the untreated group, thus underestimating the benefits derived from early treatment. Hence, the immunological and virological benefits derived from early treatment would last for at least one year after treatment interruption.

Short-course treatment during PHI was associated with better immunological and virological outcomes after treatment interruption. The net benefits of CD4 count and viral load were 162.42 cells/μl and 0.52 log copies/ml within one year after treatment interruption. This change is a clinically significant change, which may have a profound effect on HIV disease progression. An increase in set point of 0.5 log copies/ml decreases the median time to AIDS by 3 years [23], and the increased CD4 count may prolong the time until HAART has to be reinitiated because of a low CD4 count or disease progression. Theoretically, the prevention of severe loss of CD4 T cells in peripheral blood and lymphoid tissues occurring during PHI [24,25], the preservation of HIV-specific cellular and humoral immune responses [26,27], the restriction of viral diversification [28,29], and the reduced size of the HIV reservoirs [30] exerted by treatment during PHI may be the reasons explaining for these benefits.

The benefits of CD4 count and viral load derived from early treatment were not long-lasting, and may be waning over time. Viral load rebounded immediately after treatment interruption due to the existence of HIV reservoirs [31] such as infected memory CD4 T cells, macrophages and so on. It is likely that continued viral replication gradually diminished the benefits from early treatment. Yet, the accurate duration of the immunological and virological benefits needs longer follow-up in RCT trials.

As for the impact of treatment duration on the immunovirological outcomes, it showed that extended treatment duration over 12 months did not have more benefits than those treated for less than 12 months. This is consistent with the results from the randomized Primo-SHM trial [13], but is different from results of SPARTAC trial [15]. This may indicate that 12 weeks treatment is too short to have any effect, so treatment duration should be longer than 12 weeks to observe any benefit. The lack of additional immunological benefits with longer treatment duration may be explained by the fact that the CD4 cell reconstruction was fast during the first 6 months, and then it became relatively slow thereafter [32]. We could not perform meta-analysis assessing the impact of treatment initiation time on the immunovirological outcomes, because of different definitions of treatment initiation time and insufficient data. Some studies reported earlier treatment would result in more significant immunovirological benefits [20,33], but not supported by others [34], so further studies are needed.

This meta-analysis was characterized with larger sample size, more reliable conclusions, and more factors involved in this comprehensive analysis. Yet, some limitations of this study should be noted. The main one is the underestimation of the immunovirological benefits as mentioned above due to possible bias existed in original studies. Future analysis requires for more well-designed randomized controlled trials for accurate evaluation of the duration of benefits derived from early treatment. Another limitation is that subjects enrolled in this meta-analysis were adults and adolescents, so the conclusions from this study are applicable only to adults and adolescents.

In summary, compared to the untreated arm, treatment during PHI not only increased CD4 count but also lowered viral load after treatment interruption. Findings from this meta-analysis are of great significance for the clinical management of PHI. As CD4 count and viral load are strong predictors of HIV disease progression [35], early treatment possibly delays HIV disease progression for at least a period of time. Although extended follow-up studies are needed to ascertain the long-term benefits of early treatment, this adds to the evidence that supports early treatment. When considering initiating ART during PHI, the risk-benefit should be assessed in specific individuals as they are not the same. Patients’ adherence [36], possible drug toxicity [37], risk of development of drug-resistance which would affect the risk-benefit should be considered. Besides, rapid progressors would strongly benefit from early treatment. Moreover, the ethical questions raised by limitations in ART regimens, ART accessibility, and drug efficacy monitoring in developing countries should also be taken into consideration [38].

## Supporting Information

Checklist S1 **PRISMA Checklist.**
(DOC)

## References

[1] McMichael AJ, Borrow P, Tomaras GD, Goonetilleke N, Haynes BF (2010) The immune response during acute HIV-1 infection: clues for vaccine development. Nat Rev Immunol 10: 11-23. doi:10.1038/nri2674. PubMed: 20010788. 20010788 PMC3119211

[2] http://www.aidsinfo.nih.gov/contentfiles/lvguidelines/adultandadolescentgl.pdf.

[3] http://www.europeanaidsclinicalsociety.org/images/stories/eacs-pdf/eacsguidelines-v6.0-english.pdf.

[4] http://whqlibdoc.who.int/publications/2010/9789241599764_eng.pdf.

[5] Steingrover R, Garcia EF, van Valkengoed IGM, Bekker V, Bezemer D et al. (2010) Transient Lowering of the Viral Set Point After Temporary Antiretroviral Therapy of Primary HIV Type 1. Infection - AIDS Research and Human Retroviruses 26: 379-387. doi:10.1089/aid.2009.0041. 20377419

[6] Seng R, Goujard C, Desquilbet L, Sinet M, Rouzioux C et al. (2008) Rapid CD4+ cell decrease after transient cART initiated during primary HIV infection (ANRS PRIMO and SEROCO cohorts). J Acquir Immune Defic Syndr 49: 251-258. doi:10.1097/QAI.0b013e318189a739. PubMed: 18845951. 18845951

[7] Fidler S, Fox J, Touloumi G, Pantazis N, Porter K et al. (2007) Slower CD4 cell decline following cessation of a 3 month course of HAART in primary HIV infection: findings from an observational cohort. AIDS 21: 1283-1291. doi:10.1097/QAD.0b013e3280b07b5b. PubMed: 17545704. 17545704

[8] von Wyl V, Gianella S, Fischer M, Niederoest B, Kuster H et al. (2011) Early Antiretroviral Therapy During Primary HIV-1 Infection Results in a Transient Reduction of the Viral Setpoint upon Treatment Interruption. PLOS ONE 6. 10.1371/journal.pone.0027463 PMC3216952 22102898

[9] Koegl C, Wolf E, Hanhoff N, Jessen H, Schewe K et al. (2009) Treatment during primary HIV infection does not lower viral set point but improves CD4 lymphocytes in an observational cohort. Eur J Med Res 14: 277-283. PubMed: 19661009. 19661009 10.1186/2047-783X-14-7-277 PMC3458637

[10] Pantazis N, Touloumi G, Vanhems P, Gill J, Bucher HC et al. (2008) The effect of antiretroviral treatment of different durations in primary HIV infection. AIDS 22: 2441-2450. doi:10.1097/QAD.0b013e328319ea4e. PubMed: 19005267. 19005267

[11] Kinloch-De Loës S, Hirschel BJ, Hoen B, Cooper DA, Tindall B et al. (1995) A controlled trial of zidovudine in primary human immunodeficiency virus infection. N Engl J Med 333: 408-413. doi:10.1056/NEJM199508173330702. PubMed: 7616989. 7616989

[12] Hogan CM, Degruttola V, Sun X, Fiscus SA, Del Rio C et al. (2012) The setpoint study (ACTG A5217): effect of immediate versus deferred antiretroviral therapy on virologic set point in recently HIV-1-infected individuals. J Infect Dis 205: 87-96. doi:10.1093/infdis/jir699. PubMed: 22180621. 22180621 PMC3242744

[13] Grijsen ML, Steingrover R, Wit FW, Jurriaans S, Verbon A et al. (2012) No Treatment versus 24 or 60 Weeks of Antiretroviral Treatment during Primary HIV Infection: The Randomized Primo-SHM Trial. PLoS Med 9: e1001196 PubMed: 22479156. 22479156 10.1371/journal.pmed.1001196 PMC3313945

[14] Desquilbet L, Goujard C, Rouzioux C, Sinet M, Deveau C et al. (2004) Does transient HAART during primary HIV-1 infection lower the virological set-point? AIDS 18: 2361-2369. PubMed: 15622312. 15622312

[15] Fidler S, Porter K, Ewings F, Frater J, Ramjee G et al. (2013) Short-course antiretroviral therapy in primary HIV infection. N Engl J Med 368: 207-217. doi:10.1056/NEJMoa1110039. PubMed: 23323897. 23323897 PMC4131004

[16] Mingrone H, Acuipil C, Cordova E, Loiza E, Porteiro N (2010) Temporary antiretroviral therapy during primary HIV-1 infection in Argentina. XVIII. International AIDS Conference [Abstract No. CDB0152].

[17] Prazuck T, Hocqueloux L, Rouzioux C, Avettand-Fenoel V, Watier H et al. (2006) Could early and prolonged antiretroviral therapy achieve long-term «remission» in some acutely HIV-1-infected individuals? XVI. International AIDS Conference [Abstract No. CDB0025].

[18] Steingrover R, Garcia EF, van Valkengoed IG, Bekker V, Bezemer D et al. (2010) Transient lowering of the viral set point after temporary antiretroviral therapy of primary HIV type 1 infection. AIDS Res Hum Retroviruses 26: 379-387. doi:10.1089/aid.2009.0041. PubMed: 20377419. 20377419

[19] Lampe FC, Porter K, Kaldor J, Law M, Kinloch-de Loes S et al. (2007) Effect of transient antiretroviral treatment during acute HIV infection: comparison of the Quest trial results with CASCADE natural history study. Antivir Ther 12: 189-193. PubMed: 17503661. 17503661

[20] Hecht FM, Wang L, Collier A, Little S, Markowitz M et al. (2006) A multicenter observational study of the potential benefits of initiating combination antiretroviral therapy during acute HIV infection. J Infect Dis 194: 725-733. doi:10.1086/506616. PubMed: 16941337. 16941337

[21] Streeck H, Jessen H, Alter G, Teigen N, Waring MT et al. (2006) Immunological and virological impact of highly active antiretroviral therapy initiated during acute HIV-1 infection. J Infect Dis 194: 734-739. doi:10.1086/503811. PubMed: 16941338. 16941338

[22] Jansen CA, De Cuyper IM, Steingrover R, Jurriaans S, Sankatsing SU et al. (2005) Analysis of the effect of highly active antiretroviral therapy during acute HIV-1 infection on HIV-specific CD4 T cell functions. AIDS 19: 1145-1154. doi:10.1097/01.aids.0000176214.17990.94. PubMed: 15990567. 15990567

[23] Gupta SB, Jacobson LP, Margolick JB, Rinaldo CR, Phair JP et al. (2007) Estimating the benefit of an HIV-1 vaccine that reduces viral load set point. J Infect Dis 195: 546-550. doi:10.1086/510909. PubMed: 17230414. 17230414

[24] Guadalupe M, Reay E, Sankaran S, Prindiville T, Flamm J et al. (2003) Severe CD4+ T-cell depletion in gut lymphoid tissue during primary human immunodeficiency virus type 1 infection and substantial delay in restoration following highly active antiretroviral therapy. J Virol 77: 11708-11717. doi:10.1128/JVI.77.21.11708-11717.2003. PubMed: 14557656. 14557656 PMC229357

[25] Mattapallil JJ, Douek DC, Hill B, Nishimura Y, Martin M et al. (2005) Massive infection and loss of memory CD4+ T cells in multiple tissues during acute SIV infection. Nature 434: 1093-1097. doi:10.1038/nature03501. PubMed: 15793563. 15793563

[26] Oxenius A, Price DA, Easterbrook PJ, O'Callaghan CA, Kelleher AD et al. (2000) Early highly active antiretroviral therapy for acute HIV-1 infection preserves immune function of CD8+ and CD4+ T lymphocytes. Proc Natl Acad Sci U S A 97: 3382-3387. PubMed: 10737796. 10737796 10.1073/pnas.97.7.3382 PMC16248

[27] Rosenberg ES, Altfeld M, Poon SH, Phillips MN, Wilkes BM et al. (2000) Immune control of HIV-1 after early treatment of acute infection. Nature 407: 523-526. doi:10.1038/35035103. PubMed: 11029005. 11029005

[28] Chamberland A, Sylla M, Boulassel MR, Baril JG, Côté P et al. (2011) Effect of antiretroviral therapy on HIV-1 genetic evolution during acute infection. International Journal STD and Aids 22: 146-150. doi:10.1258/ijsa.2010.010292. PubMed: 21464451. 21464451

[29] Keele BF, Giorgi EE, Salazar-Gonzalez JF, Decker JM, Pham KT et al. (2008) Identification and characterization of transmitted and early founder virus envelopes in primary HIV-1 infection. Proc Natl Acad Sci U S A 105: 7552-7557. doi:10.1073/pnas.0802203105. PubMed: 18490657. 18490657 PMC2387184

[30] Strain MC, Little SJ, Daar ES, Havlir DV, Gunthard HF et al. (2005) Effect of treatment, during primary infection, on establishment and clearance of cellular reservoirs of HIV-1. J Infect Dis 191: 1410-1418. doi:10.1086/428777. PubMed: 15809898. 15809898

[31] Chun TW, Moir S, Kovacs C, Fauci AS (2011) Rebound of plasma viremia following cessation of antiretroviral therapy despite profoundly low levels of HIV reservoir: implications for eradication Reply. AIDS 25: 872-873. doi:10.1097/QAD.0b013e328344c25a. PMC3154092 20962613

[32] Lifson AR, Krantz EM, Eberly LE, Dolan MJ, Marconi VC et al. (2011) Long-term CD4+ lymphocyte response following HAART initiation in a U.S. Military prospective cohort. AIDS Res Ther 8: 2. doi:10.1186/1742-6405-8-2. PubMed: 21244701. 21244701 PMC3037838

[33] Gianella S, von Wyl V, Fischer M, Niederoest B, Battegay M et al. (2011) Effect of early antiretroviral therapy during primary HIV-1 infection on cell-associated HIV-1 DNA and plasma HIV-1 RNA. Antivir Ther 16: 535-545. doi:10.3851/IMP1776. PubMed: 21685541. 21685541

[34] Volberding P, Demeter L, Bosch RJ, Aga E, Pettinelli C et al. (2009) Antiretroviral therapy in acute and recent HIV infection: a prospective multicenter stratified trial of intentionally interrupted treatment. AIDS 23: 1987-1995. doi:10.1097/QAD.0b013e32832eb285. PubMed: 19696651. 19696651 PMC2888600

[35] Mellors JW, Muñoz A, Giorgi JV, Margolick JB, Tassoni CJ et al. (1997) Plasma viral load and CD4+ lymphocytes as prognostic markers of HIV-1 infection. Ann Intern Med 126: 946-954. doi:10.7326/0003-4819-126-12-199706150-00003. PubMed: 9182471. 9182471

[36] Sabundayo BP, McArthur JH, Langan SJ, Gallant JE, Margolick JB (2006) High frequency of highly active antiretroviral therapy modifications in patients with acute or early human immunodeficiency virus infection. Pharmacotherapy 26: 674-681. doi:10.1592/phco.26.5.674. PubMed: 16637796. 16637796

[37] Schiffer V, Deveau C, Meyer L, Iraqui I, Nguyen-Wartel A et al. (2004) Recent changes in the management of primary HIV-1 infection: results from the French PRIMO cohort. HIV Med 5: 326-333. doi:10.1111/j.1468-1293.2004.00231.x. PubMed: 15369507. 15369507

[38] Gallant JE, Mehta SH, Sugarman J (2013) Universal Antiretroviral Therapy for HIV Infection: Should US Treatment Guidelines Be Applied to Resource-Limited Settings? Clin Infect Dis 57: 884-887. doi:10.1093/cid/cit382. PubMed: 23759345. 23759345 PMC3749746

